# Ekbom Syndrome Following Cosmetic Biopolymer Injections Presenting With Neuropsychiatric Manifestations: A Case Report

**DOI:** 10.7759/cureus.107117

**Published:** 2026-04-15

**Authors:** Christian David Galindo, Maria Mercedes Uribe Isaza, Daniela Osorno Avendaño, Andres Lopez, Leidy Vanessa Fajardo

**Affiliations:** 1 Psychiatry, Corporación Universitaria Remington, Medellín, COL; 2 Psychiatry and Behavioral Sciences, Mental Antioquia Hospital, Medellín, COL; 3 Psychiatry and Behavioral Sciences, Corporación Universitaria Remington, Medellín, COL; 4 Child and Adolescent Psychiatry, Universidad El Bosque, Bogotá, COL; 5 Psychiatry and Behavioral Sciences, Fundación Universitaria de Ciencias de la Salud, Bogotá, COL

**Keywords:** biopolymer injections, delusional disorder, ekbom syndrome, neuropsychiatric manifestations, psychotic disorder, somatic delusional disorder

## Abstract

Ekbom syndrome (delusional infestation) is a rare psychotic disorder characterized by a fixed belief of parasitic infestation in the absence of objective medical evidence. Positioned at the intersection of psychiatry, dermatology, and neurology, it may arise as a primary condition or secondary to underlying medical, psychiatric, or iatrogenic factors. Emerging evidence suggests that chronic inflammatory and neuroimmune processes may contribute to altered somatic perception and the development of delusional beliefs in vulnerable individuals.

We present a case of a 55-year-old female with no prior psychiatric history who developed a progressive neuropsychiatric syndrome following cosmetic biopolymer injections in the face, hands, feet, and dorsal region, performed approximately one and a half years prior in a non-medical setting. Symptom onset was insidious, initially marked by progressive cutaneous changes, predominantly hyperpigmentation, which evolved into a persistent and disproportionate preoccupation with her bodily state. Over time, this was accompanied by a generalized anxiety syndrome characterized by psychomotor agitation, insomnia, somatic hypervigilance, muscle tension, and ruminative thoughts centered on perceived physical alterations. The clinical course culminated in the emergence of a fixed belief of infestation beneath the skin, consistent with secondary Ekbom syndrome.

Comprehensive laboratory evaluation and cross-sectional imaging excluded an identifiable organic etiology. Treatment with an antipsychotic agent combined with a selective serotonin reuptake inhibitor was initiated, alongside referral for multidisciplinary management involving dermatology and plastic surgery.

This case underscores cosmetic biopolymer exposure as a potential and underrecognized precipitating factor for secondary Ekbom syndrome, possibly mediated through chronic inflammatory responses and dysregulation of body perception. It highlights the need for early recognition of evolving neuropsychiatric symptoms in patients with prior cosmetic interventions and supports an integrated, multidisciplinary approach to prevent diagnostic delay, inappropriate treatments, and chronic progression in complex psychodermatological presentations.

## Introduction

Ekbom syndrome remains an underrecognized yet clinically significant psychotic disorder situated at the intersection of psychiatry, dermatology, and neurology [1]. According to the Diagnostic and Statistical Manual of Mental Disorders, Fifth Edition (DSM-5), it is classified within the somatic subtype of delusional disorders and is characterized by a persistent and unshakeable belief of infestation by parasites or other organisms in the absence of objective medical or microbiological evidence [2]. The intensity and chronicity of this conviction frequently result in repeated healthcare utilization, fragmented clinical pathways, and substantial delays in accurate psychiatric diagnosis and treatment [3].

Clinically, Ekbom syndrome may present as a primary monosymptomatic disorder or as a secondary condition associated with psychiatric disorders, systemic medical illnesses, neurological diseases, or substance use [4]. Reported associations include endocrine disturbances, particularly hyperthyroidism, nutritional deficiencies such as vitamin B12 and folate deficiency, neurodegenerative and cerebrovascular conditions, and chronic infectious diseases, including HIV and syphilis. Substance-induced cases, especially those related to stimulant use such as cocaine and methamphetamine, have also been well documented [5].

Epidemiologically, Ekbom syndrome is a rare condition, with incidence rates ranging from 1.9 to 27.3 cases per 100,000 person-years depending on the population studied and diagnostic setting [6]. It predominantly affects middle-aged and older adults, with a mean age of onset between 57 and 61 years, and a modest female predominance reported across clinical series [7].

The pathophysiology of Ekbom syndrome remains incompletely understood; however, converging evidence supports a central role of dopaminergic dysregulation, particularly involving striatal pathways [8]. Alterations in dopamine transporter function and increased extracellular dopamine levels have been implicated in aberrant salience attribution, a key mechanism underlying the formation and persistence of delusional beliefs, although these models are largely extrapolated from broader psychosis research.

From a clinical perspective, patients frequently report intense pruritus and abnormal cutaneous sensations, such as crawling, biting, or stinging perceptions, commonly referred to as formication [9]. These distressing experiences often lead to repetitive behaviors, including scratching, skin picking, and the application of irritant substances, resulting in secondary excoriations and self-inflicted lesions. In some cases, the delusional belief may extend to close contacts, giving rise to shared psychotic disorder and further complicating clinical management [10].

Accurate diagnosis requires a high index of suspicion and systematic exclusion of dermatological, infectious, neurological, and systemic medical conditions, together with a comprehensive psychiatric assessment. Early recognition and a multidisciplinary approach are essential to reduce patient distress, avoid unnecessary interventions, and improve clinical outcomes [11].

In this context, we present a case of Ekbom syndrome associated with cosmetic biopolymer exposure, highlighting a potential and underrecognized iatrogenic trigger and underscoring a critical gap in the current literature regarding its neuropsychiatric implications [12].

## Case presentation

A 55-year-old female with no prior psychiatric history was evaluated in the emergency department due to a progressively evolving clinical condition following biopolymer injections in the face, hands, feet, and dorsal region, performed approximately one and a half years earlier in a non-medical setting.

Symptom onset was insidious, initially characterized by the progressive development of cutaneous changes, predominantly hyperpigmentation, which led to a persistent and disproportionate concern regarding her bodily state. Over time, a generalized anxiety syndrome emerged, manifested by psychomotor agitation, difficulty with sleep initiation and maintenance, somatic hypervigilance, muscle tension, and ruminative thoughts focused on perceived physical changes, with a progressive course and tendency toward chronicity.

However, upon direct examination, no structures consistent with parasitic infestation were identified; instead, the sample consisted of inert material likely originating from cutaneous debris and environmental contaminants. Subsequent laboratory analysis confirmed that the specimen corresponded to coffee bean fragments rather than biological material.

In the emergency department, the patient was evaluated in stable general condition, hemodynamically compensated, with vital signs within normal limits. A comprehensive diagnostic workup was undertaken to exclude an underlying organic etiology. Laboratory investigations, including a complete blood count, inflammatory markers, electrolytes, and renal function tests, were within normal ranges, and infectious screening, including HIV and Venereal Disease Research Laboratory (VDRL) tests, was negative (Table 1).

**Table 1:** Relevant laboratory findings. HIV: human immunodeficiency virus; VDRL: Venereal Disease Research Laboratory.

Category	Test	Patient’s result	Reference range/Interpretation
Infectious screening	HIV serology	Negative	Negative
	VDRL	Non-reactive	Non-reactive
Electrolytes and renal function	Sodium (Na, mmol/L)	140.9	135-145
	Potassium (K, mmol/L)	3.9	3.5-5.0
	Chloride (Cl, mmol/L)	105.7	98-107
	Creatinine (mg/dL)	0.7	0.6-1.2
Inflammatory markers	C-reactive protein (mg/dL)	0.19	<0.5
Vitamin levels	Vitamin B12 (pg/mL)	500	200-900
	Folic acid (ng/mL)	8	4-20
Complete blood count	White blood cell count (cells/µL)	9,290	4,000-11,000
	Neutrophils (cells/µL)	3,530	2,000-7,000
	Lymphocytes (cells/µL)	4,520	1,000-4,000
	Hemoglobin (g/dL)	12.5	12-16
	Hematocrit (%)	36.6	36-46
	Platelets (cells/µL)	502,000	150,000-450,000

**Figure 1:**
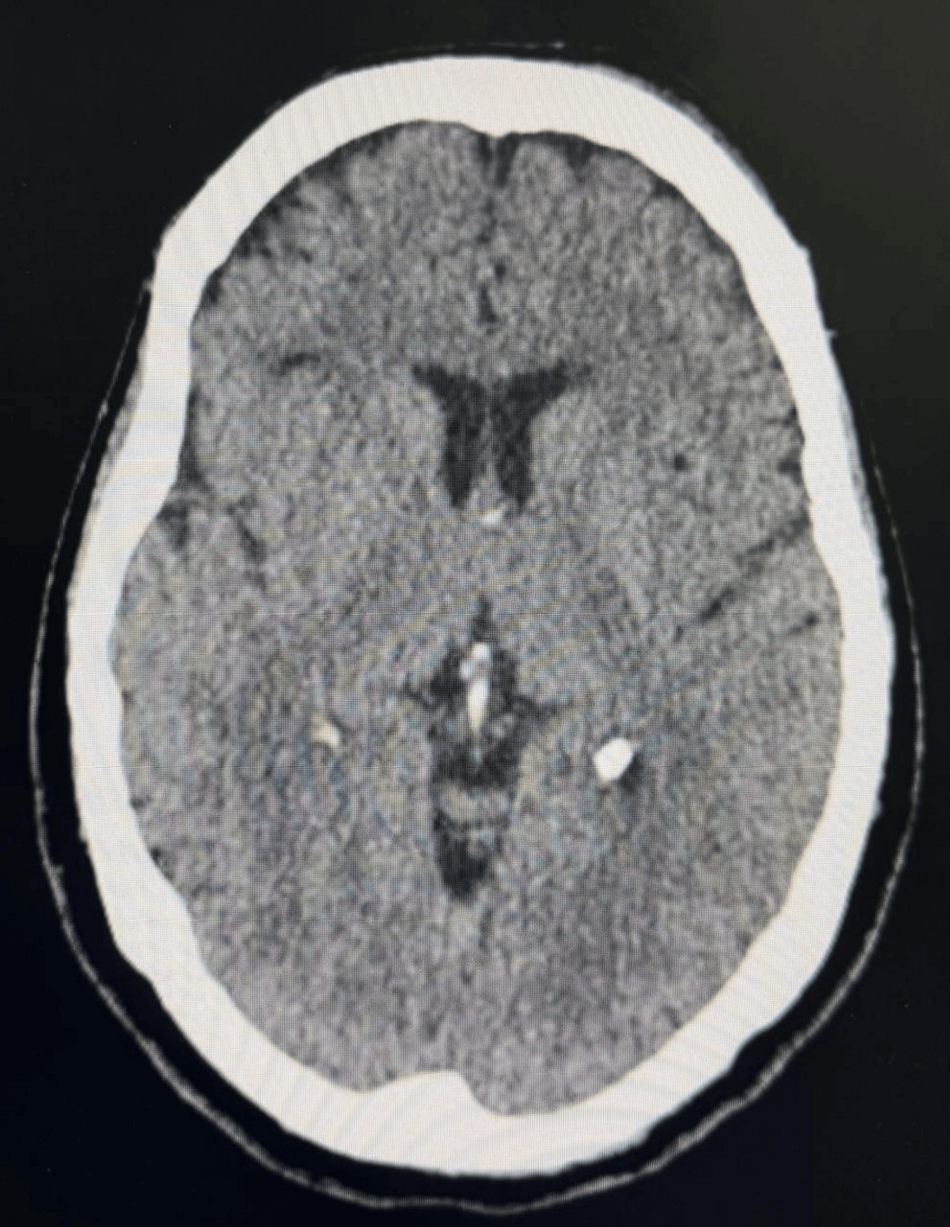
Non-contrast CT of the brain. Axial non-contrast CT scan shows preserved gray-white matter differentiation and symmetric ventricular configuration, with no signs of acute hemorrhage, mass effect, or ischemia. Despite significant neuropsychiatric manifestations, structural neuroimaging may remain unremarkable in early disease stages.

Cranial computed tomography (CT) showed no evidence of intracranial pathology (Figure 1).

**Figure 2:**
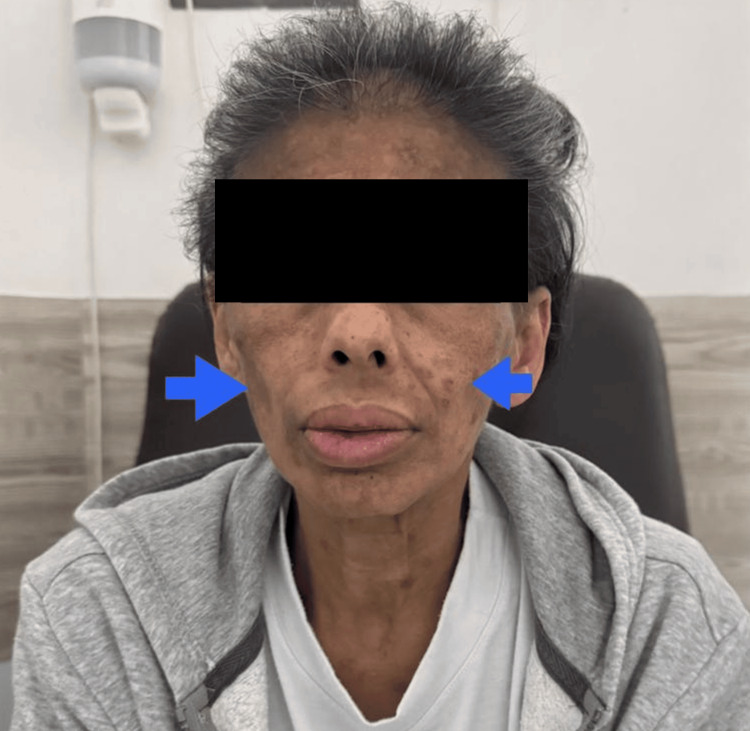
Facial cutaneous findings. Frontal view of the patient demonstrating multiple hyperpigmented macules and plaques distributed over the malar, periorbital, and frontal regions, with irregular borders and varying degrees of confluence. The lesions show a heterogeneous brownish pigmentation, consistent with post-inflammatory changes. The blue arrow indicates the hyperpigmented macules. No ulceration, vesiculation, or signs of active infection are observed.

Neurological evaluation revealed no focal deficits or signs of structural or functional central nervous system involvement, effectively excluding an organic etiology. From a dermatological perspective, multiple cutaneous lesions were documented in the facial and cervical regions, characterized by hyperpigmented macules and plaques, some of which were confluent, along with discrete subcutaneous nodular lesions of indurated appearance, non-ulcerated, and without signs of active infection (Figure 2).

**Figure 3:**
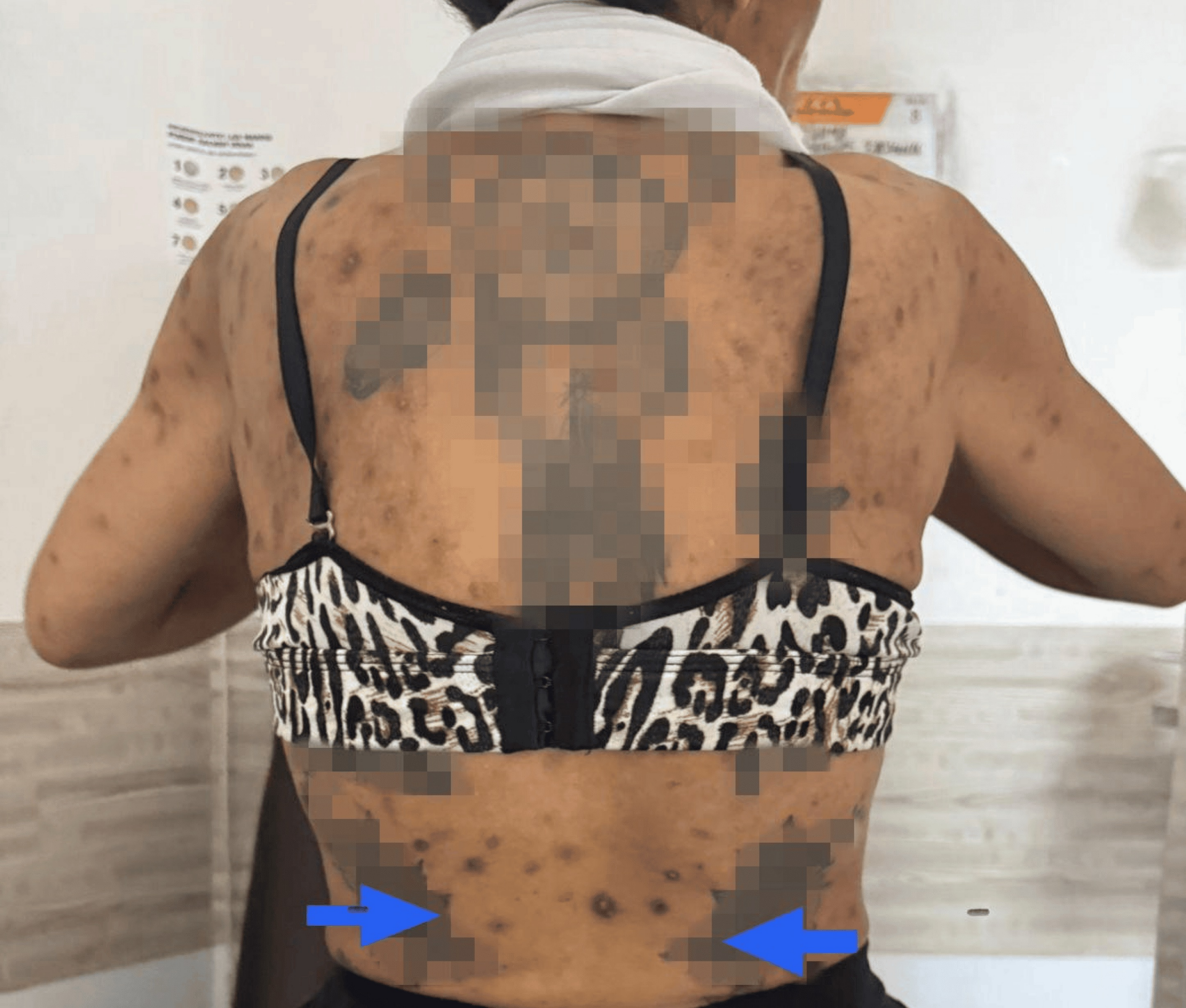
Posterior thoracic involvement. Posterior thoracic region showing widespread hyperpigmented macules and patches with irregular distribution and varying degrees of confluence, associated with superficial excoriations. Findings are consistent with post-inflammatory hyperpigmentation secondary to chronic pruritus. The blue arrow indicates the lesion. No evidence of active infection is observed.

The posterior thoracic skin demonstrated multiple areas of superficial excoriation associated with diffuse hyperpigmented macules and patches, consistent with post-inflammatory hyperpigmentation in the setting of chronic pruritus (Figure 3).

These findings were interpreted as dermatosis secondary to biopolymer injection, as confirmed by histopathological analysis obtained through skin biopsy, with no evidence of active parasitic infestation.

Psychiatric evaluation revealed preserved thought form with structured somatic delusional content, sustained with high conviction, associated with somatic hypervigilance and persistent pruritus, without evidence of auditory or visual hallucinations or active suicidal ideation. Judgment and insight were impaired.

The differential diagnosis included somatic-type delusional disorder, primary psychotic disorders, and secondary organic causes; however, the absence of clinical, laboratory, and neuroimaging findings suggestive of medical disease allowed for the exclusion of an organic etiology.

Based on interdisciplinary clinical integration, a diagnosis of somatic-type delusional disorder (Ekbom syndrome) was established. Pharmacological treatment was initiated with olanzapine at a dose of 10 mg/day orally, in combination with sertraline 50 mg/day, targeting both psychotic symptoms and the associated anxiety component. The patient was admitted for short-term observation, allowing close monitoring of psychopathological evolution and treatment response, and was subsequently transitioned to outpatient follow-up.

Following treatment initiation, the patient demonstrated a favorable clinical response. Over the course of one month, there was a marked reduction in delusional conviction, accompanied by significant improvement in anxiety, behavioral symptoms, and somatic preoccupation. After stabilization of psychotic features, olanzapine was gradually discontinued, and the patient was maintained on sertraline monotherapy. She continued regular outpatient follow-up in psychiatry, alongside coordinated interdisciplinary care with dermatology and plastic surgery.

## Discussion

Delusional infestation, also known as Ekbom syndrome, is an uncommon psychiatric condition characterized by a fixed and unshakeable belief of being infested by living organisms in the absence of objective evidence. It predominantly affects middle-aged and older women, particularly after the fifth decade of life, which is consistent with the clinical profile of the present case. Its pathophysiology is complex and multifactorial, involving the interplay between psychiatric vulnerability, psychosocial stressors, and somatic triggers [12].

The present case clearly illustrates a progressive psychopathological trajectory in which initially nonspecific anxiety symptoms and somatic concerns evolve into a structured delusional system. This pattern suggests that somatic hypervigilance and chronic pruritus may play a central role in the development of delusional infestation [13]. Unlike many previously reported cases, this case particularly highlights the role of an identifiable somatic trigger: biopolymer infiltration and the subsequent cutaneous changes. These bodily alterations likely acted not only as persistent sensory stimuli but also as facilitators of the misinterpretation of bodily sensations, ultimately contributing to the consolidation of delusional content. In this regard, this case provides clinically relevant insight into the role of iatrogenic dermatological factors as potential precipitants of somatic psychotic phenomena [14].

The presence of the so-called matchbox sign, evidenced by the patient presenting material purported to contain parasites, represents a classic feature that supports the diagnosis. This behavior reflects not only the strength of the delusional conviction but also the patient’s attempt to validate her experience within a biomedical framework [15]. As described in the literature, such material typically consists of cutaneous debris or environmental contaminants, as observed in this case, underscoring the importance of careful and objective examination.

A critical component in the evaluation of these patients is the systematic exclusion of organic causes. Chronic pruritus may be associated with a wide range of conditions, including dermatological diseases, metabolic disorders, endocrinopathies, infections, and neurological pathologies. In this case, a comprehensive diagnostic approach, including laboratory studies, neuroimaging, and multidisciplinary assessment, failed to identify any systemic metabolic, infectious, or structural cause, thereby supporting a secondary psychiatric condition precipitated by biopolymer-induced dermatosis. The absence of cognitive impairment, substance use, or neurological abnormalities further reinforced this conclusion.

The differential diagnosis includes primary psychotic disorders, body dysmorphic disorder, excoriation (skin-picking) disorder, and Morgellons disease [16]. Although repetitive skin manipulation was present, the defining feature in this case was the persistent and unshakeable belief of infestation, which distinguishes it from compulsive or anxiety-related conditions. Similarly, unlike Morgellons disease, in which patients often report inanimate materials such as fibers, this patient described living organisms, consistent with classic delusional infestation.

Management of this condition remains a significant clinical challenge. Patients frequently seek care from multiple specialties, particularly dermatology, and often resist psychiatric referral due to poor insight. In this context, establishing a strong therapeutic alliance is essential, as confrontational approaches may result in treatment refusal [17]. Psychodermatology has emerged as a particularly valuable framework, integrating dermatological and psychiatric care, including psychoeducation and cognitive-behavioral strategies, which have been shown to improve adherence and clinical outcomes.

From a pharmacological perspective, although pimozide was historically considered the treatment of choice, atypical antipsychotics have demonstrated efficacy with a more favorable safety profile. In this case, the use of olanzapine was consistent with current clinical practice and addressed both delusional symptoms and the associated anxiety component [18]. However, treatment adherence and long-term follow-up remain critical determinants of prognosis.

It is important to note that, although patients with delusional infestation are rarely aggressive toward others, they may pose a significant risk to themselves due to attempts to eliminate the perceived infestation, including excessive skin manipulation or the use of potentially harmful substances. Consequently, the functional, psychological, and social burden of the disorder can be substantial, significantly affecting quality of life and interpersonal relationships.

This case highlights the diagnostic and therapeutic complexity of delusional infestation and underscores the importance of a systematic and interdisciplinary clinical approach. Moreover, it emphasizes the potential role of somatic triggers, particularly those of iatrogenic or cosmetic origin, in the development of psychotic symptoms. Clinicians should therefore consider this diagnosis in patients presenting with persistent pruritus and infestation-related beliefs, especially in the presence of prior cutaneous alterations, promoting early and integrated intervention to optimize clinical outcomes.

## Conclusions

This case underscores the need to reconsider the role of somatic and iatrogenic factors in the pathogenesis of delusional infestation. The association between biopolymer-induced cutaneous changes and the emergence of structured somatic delusions suggests that dermatological alterations may act as key precipitating factors. A multidisciplinary approach and early intervention are essential to prevent chronicity and functional deterioration.
